# Combinatorial Polymer Electrospun Matrices Promote Physiologically-Relevant Cardiomyogenic Stem Cell Differentiation

**DOI:** 10.1371/journal.pone.0028935

**Published:** 2011-12-27

**Authors:** Mukesh K. Gupta, Joel M. Walthall, Raghav Venkataraman, Spencer W. Crowder, Dae Kwang Jung, Shann S. Yu, Tromondae K. Feaster, Xintong Wang, Todd D. Giorgio, Charles C. Hong, Franz J. Baudenbacher, Antonis K. Hatzopoulos, Hak-Joon Sung

**Affiliations:** 1 Department of Biomedical Engineering, Vanderbilt University, Nashville, Tennessee, United States of America; 2 Center for Stem Cell Biology, Vanderbilt University, Nashville, Tennessee, United States of America; 3 Department of Medicine, Division of Cardiovascular Medicine, Vanderbilt University, Nashville, Tennessee, United States of America; 4 Department of Cell and Developmental Biology, Vanderbilt University, Nashville, Tennessee, United States of America; University of Reading, United Kingdom

## Abstract

Myocardial infarction results in extensive cardiomyocyte death which can lead to fatal arrhythmias or congestive heart failure. Delivery of stem cells to repopulate damaged cardiac tissue may be an attractive and innovative solution for repairing the damaged heart. Instructive polymer scaffolds with a wide range of properties have been used extensively to direct the differentiation of stem cells. In this study, we have optimized the chemical and mechanical properties of an electrospun polymer mesh for directed differentiation of embryonic stem cells (ESCs) towards a cardiomyogenic lineage. A combinatorial polymer library was prepared by copolymerizing three distinct subunits at varying molar ratios to tune the physicochemical properties of the resulting polymer: hydrophilic polyethylene glycol (PEG), hydrophobic poly(ε-caprolactone) (PCL), and negatively-charged, carboxylated PCL (CPCL). Murine ESCs were cultured on electrospun polymeric scaffolds and their differentiation to cardiomyocytes was assessed through measurements of viability, intracellular reactive oxygen species (ROS), α-myosin heavy chain expression (α-MHC), and intracellular Ca^2+^ signaling dynamics. Interestingly, ESCs on the most compliant substrate, 4%PEG-86%PCL-10%CPCL, exhibited the highest α-MHC expression as well as the most mature Ca^2+^ signaling dynamics. To investigate the role of scaffold modulus in ESC differentiation, the scaffold fiber density was reduced by altering the electrospinning parameters. The reduced modulus was found to enhance α-MHC gene expression, and promote maturation of myocyte Ca^2+^ handling. These data indicate that ESC-derived cardiomyocyte differentiation and maturation can be promoted by tuning the mechanical and chemical properties of polymer scaffold via copolymerization and electrospinning techniques.

## Introduction

Myocardial infarction (MI) is a leading cause of death in the United States and throughout the Western world. Following MI, massive cardiomyocyte death occurs, eventually leading to the development of arrhythmias and/or congestive heart failure [1]. Myocardium is terminally differentiated tissue with limited regenerative capacity which cannot compensate for the large scale loss of cardiac tissue after MI. Currently, heart transplantation is a viable treatment method for the end stage congestive heart failure, but is not applicable for early stages of disease progression and is restricted by the limited number of donors. Cell-based therapies have therefore emerged as new potential therapeutic options for treating cardiac diseases [2].

Recently, *in situ* cellular cardiomyoplasty, a technique in which cells are delivered directly onto the hypertrophic myocardium, has shown promise as a potential strategy for myocardial regeneration following MI. Several types of donor cells have been used for this purpose, including fetal [3] and adult [4] cardiomyocytes, skeletal myoblasts [5], bone marrow derived hematopoietic stem cells [6]–[8], mesenchymal stem cells [8], [9], intrinsic cardiac stem cells [10], [11] and embryonic stem cells (ESCs) [12]–[18]. ESCs offer excellent therapeutic potential in terms of the capacity for self-renewal and the ability to differentiate into cardiomyocytes *in vitro*, thereby functionally replacing the diseased cardiac tissue [13], [14], [17]. The clinical translation of this approach, however, is limited by retention, survival and differentiation of ESCs at the injury site. For example, approximately 90% of cells are lost while circulating the vasculature or simply leak out of the injection site [19]. Additionally, the results from preclinical and clinical studies based on this method have generated inconclusive and mixed results [5], [20]–[22], indicating that the clinical translation of this approach is questionable.

An alternative therapeutic strategy to overcome these limitations is cardiac tissue engineering, a process in which cells are cultured on a natural or synthetic scaffold *in vitro* before implantation at the injury site [23]. For example, we plan to introduce the regenerated cardiac tissues at the site of injury directly attached to the matrix in a patch form. This will give the cells a foundation to adhere and grow and also minimize any inflammatory response. The properties of the scaffold can be manipulated to control cell behavior, including differentiation towards a specific lineage. The material design criteria for this type of application include (i) elasticity similar to that of native myocardium (ii) a biodegradation rate that allows for generation of new tissues, (iii) biocompatible degradation byproducts, (iv) the ability to retain and deliver cells and growth factors, (v) stabilization of cellular interactions with the myocardium, and (vi) the ability to direct differentiation of cells towards a cardiac lineage [24], [25]. ESC activity can therefore be directed by an instructive scaffold prior to implantation, thereby improving the post-operative therapeutic efficacy.

Geron Corporation (Menlo Park, CA) is currently at the forefront of regenerative medicine using embryonic stem cells for spinal cord injury [26], [27] and also has clinical trials in progress for cardiovascular remodeling. However, Geron uses proteins such as bone morphogenetic protein-4 to direct ESC differentiation. We present here the use of a selective small molecule BMP inhibitor, DMH1, based on our previous work that chemical inhibition of BMP is a robust, efficient and scalable means to induce myocardial differentiation in mouse ES cells [28].

The selection of cells and biomaterial plays an important role in tissue regeneration [29], [30]. Here, we hypothesized that polymeric biomaterial scaffolds with distinct chemical and mechanical properties could be employed to enhance the differentiation of ESCs to cardiomyocytes as a potential patch for cardiac repair. The various types of synthetic materials composed of poly(ethylene glycol), poly(lactic acid), poly(glycolic acid) and their copolymer poly(lactic-co-glycolic acid) have been applied in myocardium tissue engineering, however, the poor elasticity of these materials renders them unsuitable for myocardium patches [31]. In this study, we prepared a library of combinatorial copolymers containing different mole percentages of three components: hydrophilic polyethylene glycol (PEG), hydrophobic poly(ε-caprolactone) (PCL), and negatively-charged carboxylated-PCL (CPCL) to tune the physicochemical, mechanical, and bioactive scaffold properties for the control of ESC differentiation. Each polymer subunit was selected for the specific properties it contributes to the resulting copolymer: PCL is a semi-crystalline, biodegradable, hydrophobic polymer that has been FDA-approved in certain devices [32]; PEG is a non-toxic, biocompatible and hydrophilic polymer that reduces protein adsorption and cell attachment through steric exclusion; and CPCL facilitates cell attachment to the scaffold surface by providing a negative charge, effectively counteracting the repellant PEG effect [33]. These polymers were electrospun to create the extracellular matrix (ECM)-mimetic fiber structure and their effects on cardiac differentiation of ESCs were evaluated through a series of *in vitro* studies.

In this study, we present the effects of the physicochemical and mechanical scaffold properties on enhanced cardiac differentiation of ESCs through analysis of biochemical activities, gene and protein expression, and physiologically-relevant intracellular calcium signaling dynamics. Significant progress has been made recently in terms of developing a proper biomaterial format (e.g., injectable hydrogel and, stimuli-responsive scaffolds, and three dimensional co-culture system) [34]–[36] as a potential vehicle of delivering ESCs to dysfunctional myocardium and/or a means of promoting their cardiac differentiation. Our results provide important insight into the structure-function relationships that connect the chemical and mechanical properties of polymeric biomaterials with physiologically-relevant cardiomyogenic differentiation of ESCs using a combinatorial library of ECM-mimetic electrospun fiber scaffolds.

## Materials and Methods

### Chemicals, reagents, and polymers

ε-caprolactone and benzyl alcohol were purchased from Alfa Aesar (Ward Hill, MA, USA). Tin (II) ethyl hexanoate (Sn(Oct)_2_), benzyl alcohol, monomethoxypoly(ethylene glycol) (PEG) (M_n_ = 5000), anhydrous tetrahydrofuran (THF), lithium diisopropylamide (LDA) (2M in THF/*n*-heptane), anhydrous toluene, dichloromethane, diethyl ether were purchased from Sigma-Aldrich Chemicals (St. Louis, MO, USA); and were used as purchased unless otherwise noted. ε-caprolactone was dried and distilled over CaH_2_ immediately before polymerization. Tin (II) ethyl hexanoate was distilled under high vacuum. Benzyl alcohol was dried and distilled over CaH_2_.

Copolymers of *x* mol % PEG, *y* mol % PCL, and *z* mol % CPCL were identified as *x*%PEG-*b*-*y*%PCL-*co*-*z*%CPCL where PEG-PCL is a block copolymer but CPCL addition is random within the PCL subunit. The polymers are abbreviated *x*%PEG-*y*%PCL-*z*%CPCL.

### Synthesis of poly (ε-caprolactone) PCL and x%PEG-y%PCL diblock copolymers

PCL was synthesized through ring-opening polymerization of *ε*-caprolactone in bulk using benzyl alcohol and Sn(Oct)_2_ as the initiator and catalyst, respectively [37]. Briefly, CL (100×10^−3^ mol, 11.4 g, 10.96 mL), Sn(Oct)_2_ (100×10^−6^ mol, 40 mg), and benzyl alcohol (100×10^−6^ mol, 0.10 g, 0.10 ml) were placed in a previously flame dried, 100 mL round bottom flask equipped with three-way stopcock connected to manifold and degassed for 30 min with three freeze-pump-thaw cycles. The ampoule was immersed in an oil bath at 140°C. After 4 hour reaction, polymerization was stopped by cooling and the resulting polymer was dissolved in dichloromethane and precipitated into excess of diethyl ether. The structure was characterized by ^1^H NMR spectra. ^1^H NMR (CDCl_3_) = *δ* 4.06 (t, 3H, -OCH_2_), 2.31(t, 2H, -CH_2_), 1.66 (m, 2H, -CH_2_), 1.37 (m, 4H, -CH_2_) ppm.

**Figure 1:**
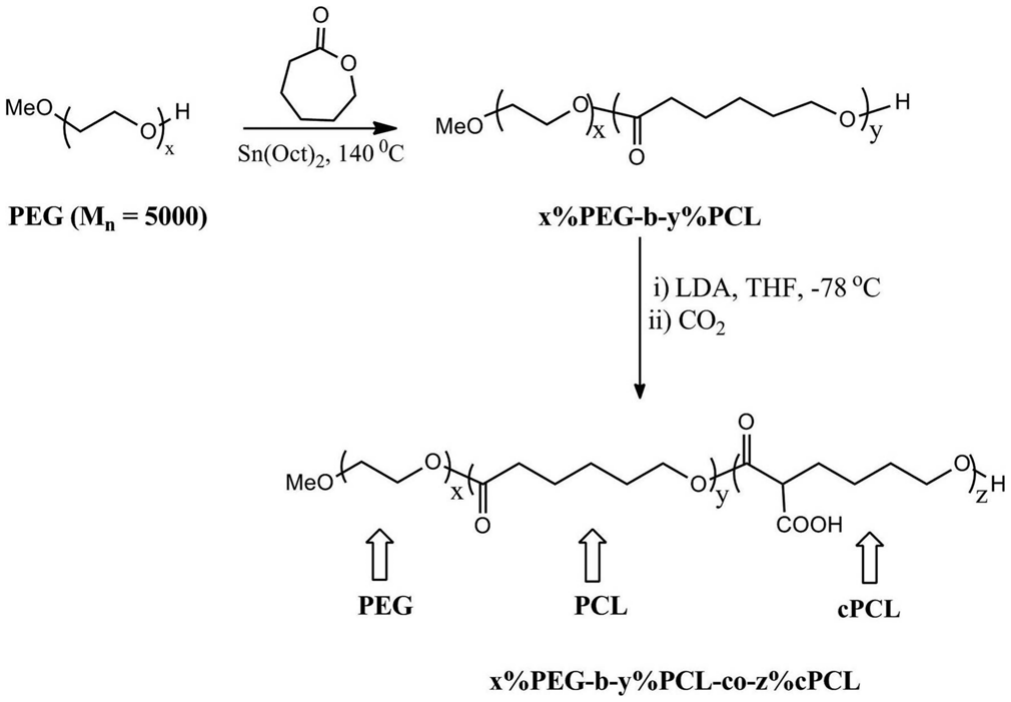
Schematic representation of polymer synthesis PEG-PCL block copolymers were first synthesized by ring-opening polymerization. CPCL was then randomly added within the PCL subunit. Polymers are abbreviated *x*%PEG-*y*%PCL-*z*%CPCL.

The same method was used to synthesize x%PEG-*b*-y%PCL diblock copolymers, except with a stoichiometric amount of 5 kDa monomethoxy-PEG in place of benzyl alcohol as the initiator (Figure 1). Polymer structure was characterized by ^1^H NMR spectra. 4%PEG-96%PCL: ^1^H NMR (CDCl_3_) = *δ* 4.06 (t, 3H, -OCH_2_), 3.65 (s, 4H, -OCH_2_), 2.31(t, 2H, -CH_2_), 1.66 (m, 2H, -CH_2_), 1.37 (m, 4H, -CH_2_) ppm. 8%PEG-92%PCL: ^1^H NMR (CDCl_3_) = *δ* 4.06 (t, 3H, -OCH_2_), 3.65 (s, 4H, -OCH_2_), 2.31(t, 2H, -CH_2_), 1.66 (m, 2H, -CH_2_), 1.37 (m, 4H, -CH_2_) ppm.

### Synthesis of 90%PCL-10%CPCL copolymer and x%PEG-y%PCL-z%CPCL terpolymers

Carboxylated PCL (CPCL) was synthesized as previously reported [37]. PCL (8.0 g, 0.067 mol) in 400 mL of anhydrous THF was added under dry nitrogen to a previously flame-dried round bottom flask. The stirred solution was placed in dry ice/acetone bath and degassed with three pump-thaw cycles. A solution of LDA, 2 M in THF/*n*-heptane (33.5 mL, 0.067 mol: 1 equivalent per monomeric unit), was added drop wise with a syringe into the reaction, which was then stirred for 30 minutes at −78°C in a bath of dry ice and acetone. A stream of dry CO_2_ gas was then generated by addition of concentrated H_2_SO_4_ to dry Na_2_CO_3_, and allowed to bubble through the solution for 30 minutes. An aqueous solution of NH_4_Cl was added to the flask to quench the reaction mixture. The resulting solution was acidified with an aqueous solution of concentrated HCl to pH 2–3. The resulting copolymer was extracted twice with 100 mL of dichloromethane. The combined organic phases were washed twice with 20 mL of distilled water and dried over anhydrous Na_2_SO_4_. After filtration, the solvent was partly evaporated under reduced pressure and the polymer was precipitated from the resulting, concentrated solution by addition of diethyl ether. The 90%PCL-10%CPCL copolymer was dried under vacuum for 24 h and stored at −20°C prior to use. ^1^H NMR (CDCl_3_) = *δ* 4.06 (t, 3H, -OCH_2_), 3.4 (m, 1H, -CH-COOH), 2.31(t, 2H, -CH_2_), 1.66 (m, 2H, -CH_2_), 1.37 (m, 2H, -CH_2_) ppm.

To synthesize x%PEG-*b*-y%PCL-*co*-z%CPCL terpolymers, the same protocol was applied, except using 4%PEG-96%PCL and 8%PEG-92%PCL in place of PCL as starting materials (Figure 1). 4%PEG-86%PCL-10%CPCL: ^1^H NMR (CDCl_3_) = *δ* 4.06 (t, 3H, -OCH_2_), 3.4 (m, 1H, - CH-COOH), 2.31(t, 2H, -CH_2_), 1.66 (m, 2H, -CH_2_), 1.37 (m, 2H, -CH_2_) ppm. 8%PEG-82%PCL-10%CPCL: ^1^H NMR (CDCl_3_) = *δ* 4.06 (t, 3H, -OCH_2_), 3.4 (m, 1H, -CH-COOH), 2.31(t, 2H, -CH_2_), 1.66 (m, 2H, -CH_2_), 1.37 (m, 2H, -CH_2_) ppm.

### Characterization of polymer synthesis

Gel permeation chromatography (GPC) was performed on a Tosoh Biosciences TSKGel SuperHZ-M mixed bed column (4×10^6^ Da exclusion limit; THF mobile phase) incubated at 40°C, with a Shimadzu SPD-10A UV detector and RID-10A refractive index detector (Shimadzu Scientific Instruments, Columbia, MD, USA), and a Wyatt miniDAWN Treos multi-angle light scattering detector (MALS; Wyatt Technology, Santa Barbara, CA, USA). Molecular weight (M_n_) and polydispersities (M_w_/M_n_) were determined against monodisperse poly(methyl methacrylate) standards (PMMA; Varian Inc., Palo Alto, CA, USA). ^1^H NMR spectra were recorded on a Brüker 400 MHz spectrometer with CDCl_3_ as solvent.

### Electrospinning and Scanning Electron Microscopy (SEM)

For electrospinning, polymer solutions (10 wt%) in a mixture of chloroform and methanol (4∶1 by volume) was loaded into a plastic syringe (10 ml) fitted with a stainless steel needle. This needle was connected to a high-voltage power supply. The solution was continuously supplied using a syringe pump at a rate of 1 ml/hour for 10 minutes (e.g. high fiber density meshes). The voltage used for electrospinning was 18 kV and the collection distance was 10 cm. The resulting fibers were collected over glass cover slips placed on a rotating mandrel at 1200 rpm. The scaffolds were dried under vacuum for 24 hours and sterilized by UV irradiation for 30 minutes before cell culture. This procedure has been proven to remove toxic organic solvents and sterilize polymer substrates effectively in previous studies [33], [38], [39].

To reduce the modulus of electrospun fibers (e.g. low fiber density meshes), the flow rate of polymer solution was reduced from 1 to 0.25 ml/hour while keeping the other conditions same. The fibers were collected over an aluminum woven wire mesh with a wire diameter of 1.0 mm, a wire spacing of 0.381 mm, and a dimension of 25 mm (*L*)×10 mm (*W*) (McMaster-Carr Co., Robbinsville NJ, USA).

For SEM imaging, electrospun scaffolds were coated with gold using a sputter coater (Cressington Scientific, Watford, United Kingdom) and their fiber structures were examined using SEM (Hitachi S-4200, Tokyo, Japan) at an accelerating voltage of 5 kV.

### Mechanical testing

Dry elastic moduli of electrospun polymer scaffolds were determined using a tabletop uniaxial testing machine (Bose ElectroForce 3100, Eden Prairie, MN, USA) using a 10-N load cell under a cross-head speed of 10 mm/min in ambient conditions (n = 3–6). Scaffold samples were prepared in a uniform rectangular form. The thicknesses of test specimens were 0.1 mm as measured using digital calipers.

Wet elastic moduli of electrospun polymer scaffolds were measured by dynamic mechanical analysis (DMA, Q800 DMA, TA Instruments, New Castle, DE, USA) (n = 3). Scaffold samples were prepared in a uniform rectangular form with the dimension of 15.0 (l)×6.6 (w) mm^2^. A wet stress and strain curve was obtained using a submersion clamp containing water at room temperature. A preload force of 0.1 N was applied to each sample and force was increased at a rate of 0.1 N/min until failure.

### ESC culture

For cell assays, mouse germ line competent CGR8 embryonic stem cells (European Collection of Cell Cultures, Salisbury, United Kingdom) were used. To monitor cardiomyogenic differentiation, CGR8 cells were stably transfected with a construct expressing the red fluorescent protein gene fused to a nuclear localization signal (DsRed-Nuc) under the α-myosin heavy chain (α-MHC) promoter. Therefore, α-MHC-expressing cells are marked with red nuclear fluorescence, allowing a visual, quantitative assessment of differentiating cardiomyocytes [40]. CGR8 ESCs were cultured in GMEM medium with 10% fetal bovine serum (FBS), 100 units/ml LIF, 2 mM L-glutamine and 50 µM β-mercaptoethanol. ESCs were grown in differentiation medium (IMDM containing 20% FBS, 0.1 mM MEM essential amino acids, 2 mM-glutamine and 100 µM β- mercaptoethanol), and maintained in 37°C under 5% CO_2_ before use.

Embryoid bodies (EBs) were formed at day 0 by inverting droplets consisting of 2.5×10^4^ ESCs/ml in media without LIF in the presence of Noggin (300 ng/mL) [41] until day 4. Lyophilized Noggin was purchased from R&D Systems (MN, USA), reconstituted with 1× Phosphate-Buffered Saline (PBS) containing 0.1% BSA as 10 mg/ml stock solutions. To confirm our findings, cardiomyogenesis was alternatively induced in EBs by administration of a selective small molecule inhibitor of BMP type-1 receptor, DMH1 (final concentration, 0.5 µM), during day 0 to 2 of differentiation [28]. Previously, we showed that a small molecule BMP inhibitor could replace Noggin in the above mentioned ESC-cardiac induction protocol [28].

After 2 days, the EBs were transferred from hanging drop culture into Petri dishes. For high fiber density substrates, EBs were moved at day 4 to electrospun polymer scaffolds coated with 0.2% gelatin on glass cover slips in tissue culture plates. For low fiber density substrates, fiber meshes were immobilized to the membrane side of modified transwell insert after removing the insert membrane. The differences in seeding methods result from the inability of low density fibers to remain effectively attached to cover slips; therefore, these meshes were held in place with the modified transwell insert. At day 10, *in vitro* measurements were performed upon visual confirmation of the presence of beating EBs.

### Intracellular Reactive Oxygen Species (ROS) and Cell Viability

Beating, α-MHC-DsRed-transfected CGR8 cells at day 10 were used to measure intracellular ROS production and cell viability. Intracellular hydrogen peroxide production was measured using dichlorofluorescein diacetate (DCFDA, Invitrogen) following established methods [38], [42]. Cell viability was measured using calcein AM (Invitrogen). The fluorescence intensity was measured on a Tecan infinite F500 plate reader (Männedorf, Switzerland) and normalized to the corresponding cell number measured from Hoechst nuclear staining.

### Gene and protein expression of α-MHC

To measure α-MHC expression, total RNA was extracted from ESCs using the RNeasy Mini Kit (Qiagen, Valencia, CA) according to the manufacturer's instructions and subsequently treated with RNase-free DNase I (Qiagen). Equal concentrations of RNA were then reverse-transcribed using the iScript cDNA synthesis kit (Bio-Rad). Real time-PCR was performed on the Bio-Rad iCycler iQ (Hercules, CA) using the iQ SYBR Green Supermix (Bio-Rad) (n = 3) and the following primers (all purchased from Sigma-Aldrich): GAPDH forward 5′-CTCACTCAAGATTGTCAGCAATG-3′ and GAPDH reverse 5′-GAGGGAGATGCTCAGTGTTGG-3′; α-MHC forward 5′-TACACTCTTCTCTACCTATGCTTCT-3′ and α-MHC reverse 5′-CACTATCTTCTTGAACTCAATGC-3′. Each primer pair was tested and its melt curve was analyzed to ensure that only a single amplicon was generated. Each test sample was assayed for target gene or GAPDH (reference gene) and the average value was used as CT. To perform statistical analyses and for graphical representation, target gene C_T_ values (A) and GAPDH C_T_ values (B) were both expressed as exponents of 2, and data represented as the ratio of 2^A^/2^B^, or 2^(A–B)^.

To measure α-MHC protein expression, the red fluorescence intensity of ESCs transfected with the α-MHC-DsRed fusion was measured through image analysis (n = 4). The fluorescence intensity was normalized to the corresponding cell number measured from Hoechst nucleus staining.

### Intracellular Calcium dynamics

EBs were mechanically detached from their polymer substrates and re-suspended in 2 ml culture media. EBs were then incubated for 15 minutes with 5 µM of the cell-permeant Ca^2+^ sensitive fluorophore Fura-2 AM (Invitrogen). EBs were then resuspended in fresh, dye-free media for 15 minutes to allow for de-esterification of the acetoxymethyl ester (AM) dye molecule.

In order to record Ca^2+^ transients, each EB was field-stimulated at 1 Hz in a custom built imaging dish using platinum wire electrodes. Excitation light was multiplexed at wavelengths of 360 nm and 380 nm using a computer controlled monochromator (Cairn, UK). Resulting fluorescence was recorded at a wavelength of 510±20 nm using an array of optical fibers positioned in the focal plane of a Zeiss Axiovert 200 microscope (Oberkochen, Germany) coupled to photomultiplier tubes (Hamamatsu Photonics, Shizuoka, Japan). Recordings were taken at multiple sites on each EB. Computed fluorescence ratios (I_360 nm_/I_380 nm_) were post-processed with a 200 Hz low-pass filter. The transient amplitude was defined as the difference between the systolic and diastolic fluorescence ratios. The decay constant was computed by fitting a single exponential decay starting from the maximum fluorescence ratio using Origin (OriginLab, MA, USA).

### Immunohistochemistry

At day 11, EBs were fixed with 2% paraformaldehyde and permeabilized with 0.2% Triton X-100. Cells were blocked with 5% bovine serum albumin in PBS for 30 minutes at 37°C. Cells were then incubated with Rabbit anti-mouse SERCA2a (Sarcoplasmic Reticulum Ca^2+^ ATPase isoform 2a) IgG antibody (provided by Dr. Sabine Huke at Vanderbilt University Medical Center) in 1∶1000 dilution with 5% bovine serum albumin in PBS for 1.5 hours at 37°C, followed by incubation with secondary FITC-conjugated goat anti-rabbit IgG antibodies (Sigma-Aldrich). The cells were imaged under a Nikon Eclipse Ti inverted fluorescence microscope (Nikon Instruments Inc, Melville, NY).

### Statistical Analysis

In all experiments, results are presented as means ± standard error mean (SEM). Comparisons between individual sample groups were performed using an unpaired Student's t-test. For all statistics, p<0.05 was considered statistically significant.

## Results

### Polymer synthesis and characterization

The test polymers with the general formula *x*%PEG-*y*%PCL-*z*%CPCL were synthesized (Figure 1) [37], [43]. The carboxyl groups in CPCL were introduced into the PCL chain by anionic activation of the α-methylene proton by LDA followed by further reaction with carbon dioxide (CO_2_) [37]. Characterization of the resulting polymers by GPC revealed that the M_n_ ranged from 65–112.5 kDa, relative to monodisperse PMMA standards (Table 1).

**Table 1:** Characterization of polymer properties.

Polymer	M_n_ a(Da)	PDI	Dry modulus^b^(MPa)	Wet modulus^c^(MPa)
**PCL**	91,720	1.25	7.58±1.0	0.79±0.18
**4%PEG-96%PCL**	93,070	1.24	21.29±3.21	0.81±0.01
**8%PEG-92%PCL**	104,200	1.21	15.40±0.77	0.74±0.04
**90%PCL-10%CPCL**	112,800	1.06	13.33±2.79	0.98±0.04
**4%PEG-86%PCL-10%CPCL**	108,400	1.16	18.11±2.46	0.71±0.04
**8%PEG-82%PCL-10%CPCL**	65,350	1.27	23.21±6.38	0.81±0.02

M_n_ was measured by GPC according to dn/dc light scattering values. Wet and dry moduli were calculated from stress/strain measurements.

a Molecular weight measured by GPC in THF,

b Measured on a uniaxial Bose ElectroForce 3100 mechanical tester,

c Measured by DMA.

Polymers were electrospun over glass cover slips to form fiber networks. The diameter, morphology and alignment of fibers were kept constant by applying similar electrospinning conditions [44]. The average diameter of fibers was found to be 0.5 µm. Table 1 shows dry and wet elastic moduli of electrospun polymer meshes. The polymers exhibited dry elastic moduli ranging from 7.58±1.0 to 23.21±6.38 MPa. Among all scaffolds tested, PCL exhibited the lowest dry elastic modulus (7.58±1.0 MPa), whereas the stiffest material was the 4%PEG-86%PCL-10%CPCL terpolymer (23.21±6.38 MPa). Following hydration to equilibrium, PEG- and CPCL-containing polymers exhibited a greater decrease in wet elastic modulus compared to PCL. Because CPCL and PEG subunits are hydrophilic, segments containing these moieties became less rigid upon hydration, causing a large decrease in wet elastic modulus.

### Maintenance of ESC Viability on Electrospun Fiber Scaffolds and Differentiation to Cardiomyocytes

To evaluate ESC viability and their differentiation into cardiomyocytes on fiber scaffolds, EBs [28] were induced to differentiate towards cardiomyocytes by Noggin treatment and grown on scaffolds for six days. Upon visual confirmation of contracting cells, cell viability, intracellular ROS levels, α-MHC expression at the gene and protein levels, and Ca^2+^ ion dynamics were measured.

**Figure 2:**
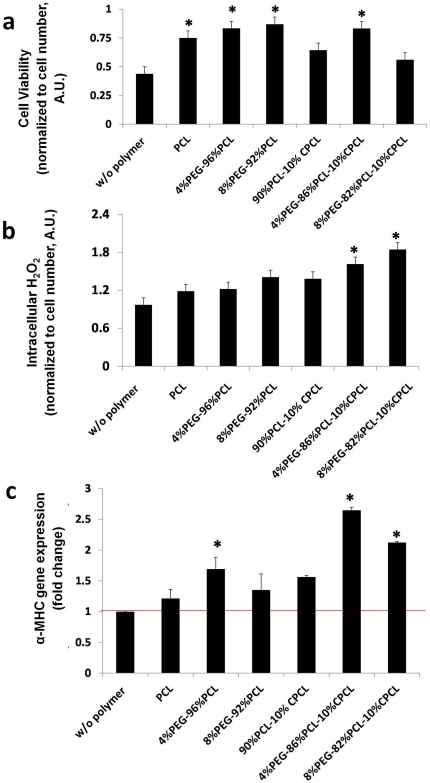
Biochemical activities of EBs on polymer scaffolds. Electrospun polymer scaffolds were shown to enhance cardiac-related biochemical activities of EBs, compared to control. (**a**) Cell viability, (**b**) intracellular H_2_O_2_, and (**c**) α-MHC gene expression. * *p*<0.05 versus control.

EBs on all test polymer types showed higher cell viability compared to control (glass coverslip without polymer, Figure 2a). In particular, PCL, 4%PEG-96%PCL, 8%PEG-92%PCL and 4%PEG-86%PCL-10%CPCL exhibited the most significant enhancement in cell viability. Since intracellular ROS have been implicated in ESC differentiation towards both cardiomyogenic and vascular cell lineages [45]–[47], we measured intracellular hydrogen peroxide (H_2_O_2_) and found that test polymer types differentially regulated intracellular H_2_O_2_ production (Figure 2b). In particular, the terpolymer types (i.e., 4%PEG-86%PCL-10%CPCL and 8%PEG-82%PCL-10%CPCL) enhanced intracellular H_2_O_2_ most significantly.

**Figure 3:**
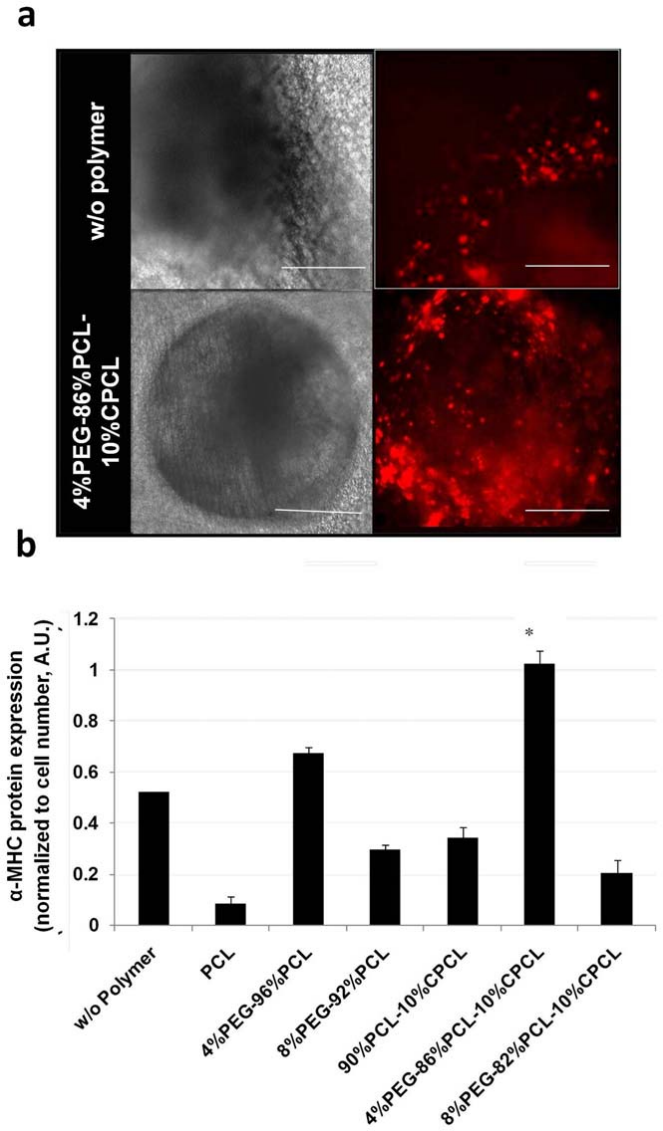
EB attachment and protein expression. (**a**) Phase contrast and fluorescence images of EBs are shown at day 10. Cells were cultured on gelatin coated glass cover slips with and without 4%PEG-86%PCL-10%CPCL copolymer scaffolds. Cells grown on 4%PEG-86%PCL-10%CPCL maintained more adhered, circular EBs and (**b**) exhibited higher α-MHC protein expression. Scale bars = 10 µm. * *p*<0.0005 versus control.

To further quantify the differentiation of ESCs to cardiomyocytes, gene expression of α-MHC, a marker of the cardiac lineage, was measured with real time PCR (Figure 2c). EBs on all test polymer types up-regulated α-MHC gene expression compared to control. EBs grown on terpolymer fiber scaffolds (i.e., 4%PEG-86%PCL-10%CPCL and 8%PEG-82%PCL-10%CPCL) exhibited the greatest increase in α-MHC gene expression (∼2-fold relative to control). To further confirm α-MHC expression, transfected EBs on fiber scaffolds were imaged and the overall fluorescence intensity from the α-MHC promoter was quantified through image analysis (Figure 3). Representative phase contrast and fluorescence images demonstrate improved attachment of EBs on 4%PEG-86%PCL-10%CPCL relative to control (Figure 3a). EBs on the 4%PEG-86%PCL-10%CPCL scaffolds also exhibited faster beating rates compared to control (data not shown). Interestingly, the level of α-MHC-related fluorescence in EBs was dependent on the polymer composition (Figure 3b). EBs cultured on 4%PEG-96%PCL and 4%PEG-86%PCL-10%CPCL scaffolds exhibited up-regulated α-MHC expression relative to control whereas protein expression was substantially down-regulated in the other test polymer groups.

Taken together, 4%PEG-86%PCL-10%CPCL was found to be the most favorable polymer composition for maintaining EB viability and enhancing their differentiation to cardiomyocytes, whereas 100%PCL was found to be the least favorable composition for the cellular activities tested.

### Effects of Scaffold Mechanical Properties on ESC Differentiation

**Figure 4:**
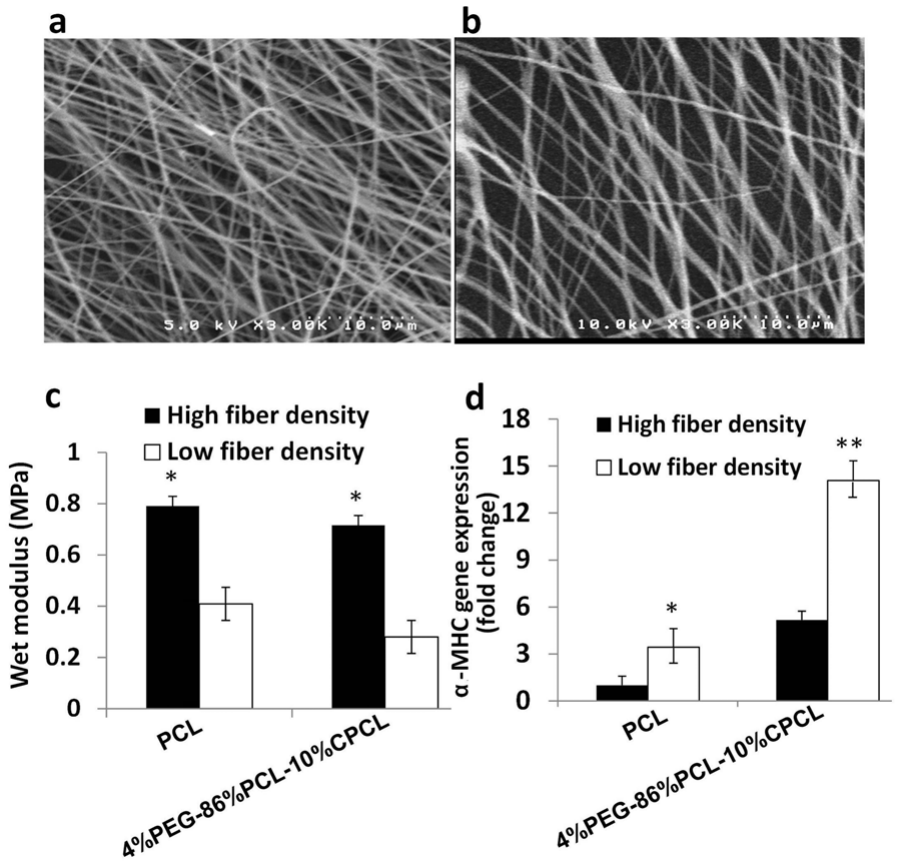
Effect of mechanical properties on EB differentiation. (**a**) SEM images of (**a**) high fiber density and (**b**) low fiber density scaffolds. (**c**) Wet modulus of PCL and 4%PEG-86%PCL-10%CPCL. * *p*<0.05 versus high density fibers. (**d**) α-MHC gene expression is enhanced on low versus high density fiber scaffolds, indicating improved differentiation. * *p*<0.05 versus high density PCL **p<0.0005 versus high density 4%PEG-86%PCL-10%CPCL.

Mechanical properties of a polymer substrate can be tuned by changing electrospinning parameters [44]. Two groups of fiber scaffolds with different fiber densities were prepared: the first was prepared by spinning at a flow rate of 1 ml/hour for 10 min, and the second was prepared by reducing the flow rate (0.25 ml/hour) and doubling the deposition time (20 minutes) to reduce fiber density. The orientation could change due to the reduced flow rate of electrospun polymer solution as the less polymer solution flows towards the rotting mandrel [44]. However, the results from assessing the fiber orientation did not show any significance difference between the low and high fiber density groups (data now shown). Due to the resulting scaffold morphology by SEM, these two scaffold types will be referred to as ‘high’ (Figure 4a) and ‘low’ (Figure 4b) fiber density scaffolds, respectively.

Scaffold fiber density proportionally correlated with wet elastic modulus (Figure 4c). PCL scaffolds with high and low fiber densities exhibited wet moduli of 0.79 MPa and 0.42 MPa, respectively. 4%PEG-86%PCL-10%CPCL scaffolds of high and low fiber densities exhibited lower moduli than PCL (0.71 and 0.28 MPa, respectively). This is likely due to the hydrophilicity of PEG and CPCL in 4%PEG-86%PCL-10%CPCL which resulted in increased hydration and water retention relative to hydrophobic PCL. As a consequence, polymer chains within the terpolymer scaffolds were better hydrated and separated more freely in an aqueous environment, leading to lower moduli.

To evaluate the effects of fiber density on EB differentiation at the gene level, EBs were cultured on two polymers types (PCL and 4%PEG-86%PCL-10%CPCL) at low and high fiber densities and α-MHC gene expression was measured. Cardiomyogenic differentiation of EBs was shown to be significantly influenced by scaffold mechanical properties (Figure 4d). For both polymer types tested, low fiber density scaffolds with decreased moduli promoted higher α-MHC gene expression than high fiber density scaffolds of the same material (2-fold higher for PCL, ∼3–4-fold higher for 4%PEG-86%PCL-10%CPCL). These results indicate that more elastic substrates enhance the differentiation of EBs into cardiomyocytes in the presence of Noggin.

### Intracellular Calcium Signaling Dynamics

**Figure 5:**
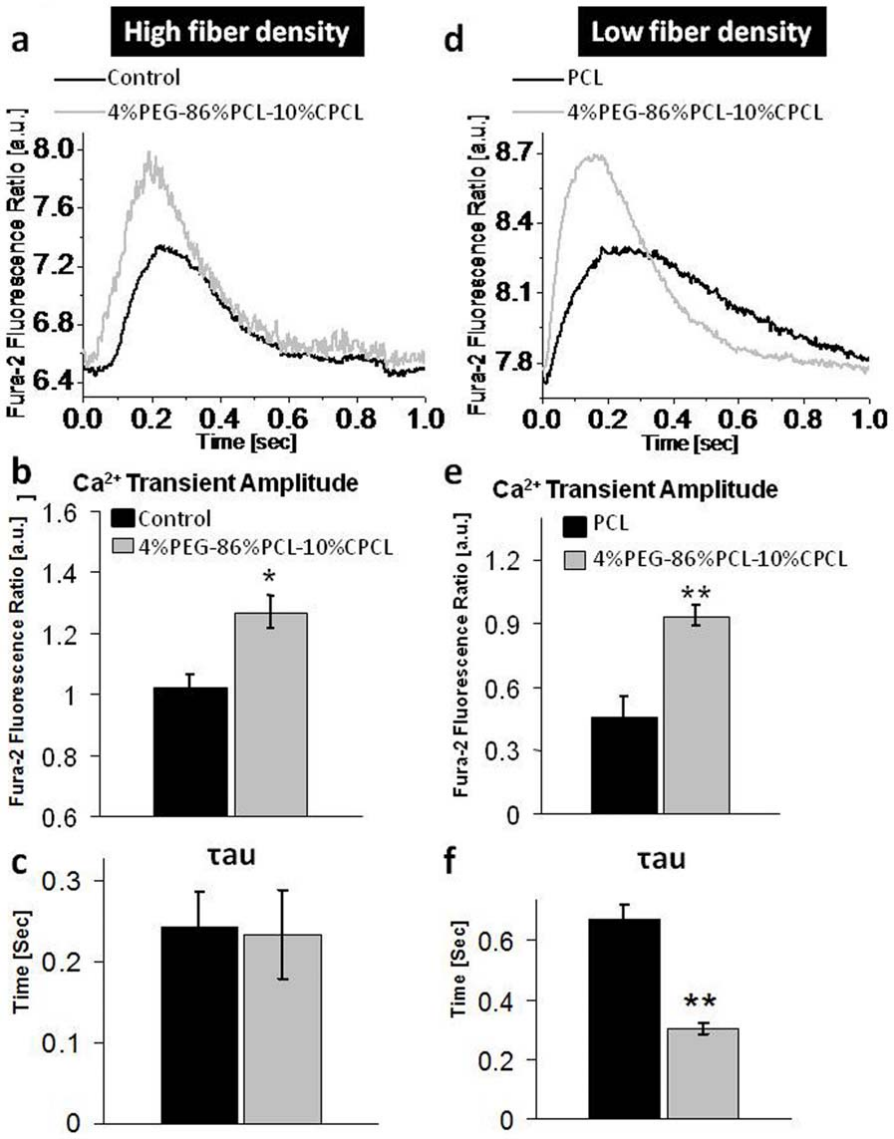
Polymer substrate elasticity affects Ca^2+^ dynamics of EBs. Ca^2+^ transients were recorded from isolated EBs from polymer scaffolds using the fluorescent ratiometric calcium dye Fura-2. EBs were subjected to 1 Hz field-stimulations during recordings. (**a, d**) Representative fluorescence traces showing the calcium transient accompanying a electrically stimulated single contraction. (**b, e**) Ca^2+^ transient amplitude, measured as the difference between diastolic and systolic Ca fluorescence intensities. (**c, f**) Decay constant of the Ca^2+^ transient, calculated by fitting the decay to a single exponential decay function. *p<0.05 and ** *p*<0.0005.

Ca^2+^ handling in cardiomyocytes constitutes a well-defined sequence of events. Ca^2+^ influx into the cells via the voltage gated L-type Ca^2+^ channel provides a trigger to release an increased amount of Ca^2+^ from the sarcoplasmic reticulum (SR) Ca^2+^ store through Ca^2+^-sensitive ryanodine receptors (RyRs). The Ca^2+^ binds to troponins and initiates contraction before it is taken up by the SR Ca^2+^ ATPase (SERCA) or transported across the sarcolemma membrane by the sodium calcium exchanger (NCX) [48]. To further validate the ability of fiber mesh scaffolds to enhance the differentiation of EBs into cardiomyocytes, intracellular Ca^2+^ transients were recorded from isolated EBs from scaffolds of differing compositions and densities. Because low density fiber scaffolds were shown to enhance α-MHC expression more effectively than high fiber density scaffolds, we compared PCL versus 4%PEG-86%PCL-10%CPCL in a low density format and PCL versus glass in a high density format (Figure 5). Due to potential different dye loading conditions of low and high density fiber samples, differences in calcium dynamics between the two types of samples were not directly compared.

The ratio of the fluorescence emission intensities of the Fura-2 fluorophore recorded from EBs at 360 and 380 nm correlates directly to the absolute intracellular Ca^2+^ concentration. By recording Fura-2 fluorescence ratios during a train of 1 Hz field stimulation, the calcium transients corresponding to individual contractions were obtained.

EBs from high fiber density 4%PEG-86%PCL-10%CPCL scaffolds were compared to control (EBs cultured on glass without polymers, Figure 5a–c). Because low fiber density scaffolds were shown to enhance α-MHC expression relative to high density scaffolds, the two polymer types were compared in a low fiber density format. For measurements of the intracellular calcium dynamics (Figure 5d–f), the ratio of the fluorescence intensities of the Fura-2 fluorophore were correlated to intracellular Ca^2+^. High fiber density 4%PEG-86%PCL-10%CPCL meshes resulted in a larger Ca^2+^ transient amplitude when compared to control (glass only, Figure 5a, b), and low fiber density 4%PEG-86%PCL-10%CPCL meshes promoted a larger Ca^2+^ transient amplitude (Figure 5 d, e) and a faster decay time τ, indicative of SERCA activity, when compared to PCL (Figure 5f). Regardless of polymer composition, low fiber density substrates enhanced Ca^2+^ transient amplitude more effectively than high fiber density 4%PEG-86%PCL-10%CPCL, indicating the strong influence of fiber density and mechanical properties on cardiomyogenic differentiation in the presence of Noggin.

For both low and high fiber density substrates, 4%PEG-96%PCL-10%CPCL fiber meshes promoted enhanced Ca^2+^ cycling, which indicates that EBs cultured on this polymer result in more ESC-derived cardiomyocytes compared to PCL only or glass control. These data suggest that the EBs isolated from the terpolymer scaffolds possess a propensity for improved Ca^2+^ handling, indicating superior cardiac function.

### DMH1 Treatment and SERCA2 expression

**Figure 6:**
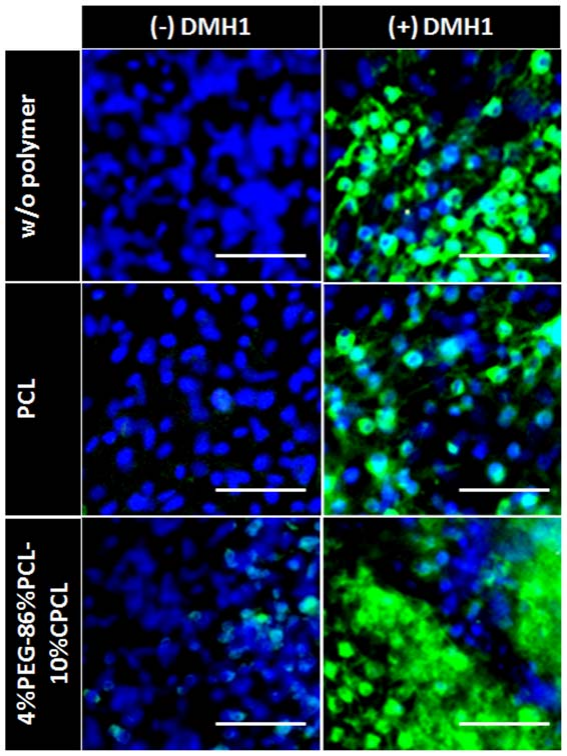
EB differentiation on polymer fiber scaffolds in the presence of DMH1. EBs with and without DMH1 treatment were stained for SERCA2a, an indicator of active Ca^2+^ transient. EBs on 4%PEG-86%PCL-10%CPCL exhibited enhanced expression of SERCA2a, compared to untreated EBs on PCL scaffolds or glass alone. Scale bars = 50 µm.

We previously showed that dorsomorphin, a small molecule inhibitor of BMP type-I receptor promotes differentiation of murine ESCs into cardiomyocytes by at least 20-fold, at the expense of other mesodermal tissues [28]. Here, we used a more selective BMP inhibitor DMH1 to promote cardiomyogenesis in ES cell. When EBs were differentiated without DMH1, there was minimal induction of cardiomyogenesis [28]. Correspondingly, when ES cells were differentiated in the absence of DMH1 and were stained for SERCA2, there was minimal expression (Figure 6). Thus, although it is possible that increased SECRA2 expression following DMH1 treatment is partially from non-cardiac sources, our results suggest strongly that increased SERCA2 expression originates from cardiomyocytes induced by DMH1 treatment. Since efficient calcium sequestration into the sarcoplasmic reticulum by SERCA2 is a feature of functional cardiomyocyte (Figure 5f), our data suggests that little or no functional cardiomyocytes form during traditional EB differentiation protocol, although low SERCA2 expression was observed on 4%PEG-86%PCL-10%CPCL (Figure 6). By contrast, EBs induced to cardiomyogenic differentiation by DMH1 demonstrated substantial SERCA2 expression even without polymers (Figure 6). Importantly, while there was no increase in SERCA2 expression on PCL, DMH1- treated EBs grown on low fiber density 4%PEG-86%PCL-10%CPCL showed markedly increased SERCA2 expression (Figure 6). These results further support low fiber density 4%PEG-86%PCL-10%CPCL as the preferred substrate for enhanced differentiation of ESCs to mature cardiomyocytes with functional excitation contraction coupling.

## Discussion

ESCs are promising therapeutic candidates in regenerative medicine due to their pluripotency and capacity for self-renewal. In recent years, much progress has been made to understand the microenvironmental cues that control the differentiation of ESCs towards particular lineages [45]–[48]. Precise guidance of ESC behavior is mandatory for clinical translation of ESC-based therapies to become a reality. Insights into structure-function relationships between the microenvironment and ESCs assist in the design of synthetic scaffolds that more effectively control ESC differentiation. For example, polymeric biomaterials can be designed to mimic a 3D extracellular matrix (ECM) network that facilitates the maintenance of cell homeostasis, phenotype, and behavior [49]. The recapitulation of such cues and signals in a 3D polymer structure is possible by tuning the desired chemical and mechanical properties through copolymerization techniques. The results presented here indicate that polymer scaffold composition and mechanical properties both play central roles in EB formation, development and their subsequent differentiation into cardiomyocytes.

To investigate the influence of scaffold properties on ESC behavior, a library of polymers (*x*%PEG-*y*%PCL-*z*%CPCL) was synthesized, characterized, and prepared as fiber substrates by electrospinning to mimic the ECM network. Each polymeric component was selected for the specific contributions made to the chemical and mechanical properties of the resulting copolymer: PCL is a semi-crystalline, hydrophobic polymer that exhibits slow degradation kinetics with biocompatible byproducts; PEG is a hydrophilic polymer that absorbs water and repels nonspecific protein adsorption through steric exclusion [33]; CPCL further promotes water absorption and facilitates cell attachment by providing a negative charge at the surface. Therefore, the presence of CPCL is expected to buffer the repellent character of PEG while simultaneously increasing hydrophilicity of the resulting material [33]. PCL was selected as the primary component of all polymer types tested because we have previously demonstrated it to be ideal for cardiovascular applications [50], [51].

The physical properties of these scaffolds were first evaluated for their applicability for cardiac regeneration. The modulus of native rat myocardium ranges from about 10 to 150 kPa [31], [52], [53]. The differentiation of cardiomyocytes from cardiac stem cells on a square grid poly *L*-(lactic acid) scaffold with a Young's modulus of about 300 kPa was successfully demonstrated [54]. Because the wet moduli of all scaffolds fell within the same order of magnitude, the electrospun test polymers were predicted to be ideal for this application. We therefore conducted a series of *in vitro* tests to evaluate enhancement of ESC cardiomyogenic differentiation on polymer substrates. Eventually, 4%PEG-86%PCL-10%CPCL was selected as an ideal chemical composition among the test polymers. Moreover, the wet modulus of 4%PEG-86%PCL-10%CPCL low density fibers showed a modulus around 280 kPa, which is similar to myocardium modulus, and our results demonstrated the promoted differentiation of cardiomyocytes from mouse ESCs on this type of fibers.

Copolymerization techniques enabled us to evaluate the effects of scaffold composition and the resulting mechanical properties on ESC differentiation. Compared to EBs grown on control glass slides, EBs grown on all test scaffolds exhibited enhanced adherence and viability, elevated intracellular H_2_O_2_ production, and increased α-MHC expression at the gene and protein levels—all of which indicate differentiation towards a cardiomyogenic lineage [45]–[47]. The slight elevation in intracellular H_2_O_2_ in EBs cultured on 4%PEG-86%PCL-10%CPCL represents healthy redox signaling that promotes differentiation without excessive, pathogenic overproduction of ROS [47]. While other polymers also caused an increase in intracellular H_2_O_2_ without a loss of cell viability (Figures 2), only the 4%PEG-86%PCL-10%CPCL terpolymer was able to promote a concurrent up-regulation of α-MHC gene and protein expression. Based on these data, we selected this particular polymer composition as the optimal formulation for enhancing EB differentiation for the remainder of the studies. In contrast, PCL fiber meshes demonstrated the weakest ability (closest to control) to enhance intracellular H_2_O_2_ production and α-MHC expression relative to other test polymer types; therefore, PCL was selected as the most suitable polymer control for the remainder of experiments.

Because substrate elasticity has been shown to tightly regulate stem cell differentiation, we evaluated the effects of scaffold mechanical properties on cardiomyogenic differentiation [55], [56]. To tune the scaffold mechanical properties, fiber scaffolds were deposited at high and low fiber densities by altering electrospinning parameters. The diameter, morphology and alignment of both fiber scaffold types were kept constant by applying similar electrospinning conditions. Compared to high fiber density scaffolds, EBs cultured on low fiber density 4%PEG-86%PCL-10%CPCL scaffolds exhibited enhanced α-MHC gene expression, indicating that a compliant substrate of the same chemical composition more effectively enhances cardiac differentiation in the presence of Noggin. We then hypothesized that this same scaffold would promote the most functional ESC-derived cardiomyocytes that exhibit calcium signaling dynamics that are characteristic of ventricular excitation contraction coupling. Indeed, this was verified by measurements of Ca^2+^ transients which revealed a physiological time course for the de- and re-polarization and functional cardiac excitation contraction coupling.

These findings were further supported by monitoring the expression of SERCA2a, a protein involved in Ca^2+^ transport. In the absence of DMH1, an inhibitor of the BMP signaling pathway, only EBs cultured on low fiber density 4%PEG-86%PCL-10%CPCL scaffolds exhibited a recognizable level of SERCA2a expression (Figure 6). Upon treatment with DMH1, EBs on all substrates expressed SERCA2a, but those cultured on 4%PEG-86%PCL-10%CPCL demonstrated the most significant up-regulation (Figure 6). These data indicate that low fiber density 4%PEG-86%PCL-10%CPCL scaffolds optimally enhanced the physiologically-relevant cardiomyogenic differentiation of ESCs.

Matrix stiffness directs stem cell differentiation into particular lineages on substrates with elasticity similar to respective native tissue [56]. In our study, the wet modulus of 4%PEG-86%PCL-90%CPCL low density fibers was about 280 kPa, which was relatively close to the modulus of the native myocardium [31], [52], [53] and induced the highest cardiomyocyte differentiation compared to stiffer substrates (Figure 3,4 and 6). Stem cells sense the rigidity of substrate and are expected to have elevated levels of phosphorylated focal-adhesion kinases when cultured on stiffer scaffolds [56], [57], which in turn promote the proliferation or migration of stem cells and inhibit the cardiomyocyte differentiation [58]. Moreover, the introduction of high porosity by low density fibers not only decreased the elasticity, but also contributed more accessible surface area to cells [59]. Thus the 4%PEG-PCL-CPCL low density fibers led to enhanced cardiogenesis as a result of combined low elasticity and high porosity.

Our findings demonstrate that the augmented differentiation of ESCs into healthy and electrophysiologically-functional cardiomyocytes relies heavily upon scaffold composition and mechanical properties. Through scaffold preparation by electrospinning, we have identified low fiber density 4%PEG-86%PCL-10%CPCL as the best substrate for enhanced cardiomyogenic differentiation of ESCs in the presence of the BMP inhibitor Noggin or DMH1. We have deduced that a more compliant substrate effectively promotes EB adhesion and cardiomyogenic differentiation, as verified by changes in gene and protein expression, biochemical activities, and Ca^2+^ signaling dynamics.

This approach for cardiac tissue engineering further supports the development of material-based guidance of ESC differentiation through elucidation of cell-matrix interactions. Our findings provide additional insight into the development of instructive matrices for enhanced post-operative differentiation, an understanding that is essential for the clinical translation of ESCs for cardiac repair. The advantages of our approach include scalability, reproducibility, low cost, and simplicity of fabrication. Additionally, the electrophysiological behavior of differentiated cardiomyocytes using this approach was similar to those described by others using alternative methods, thereby further supporting the utility of this approach as a feasible, clinically-relevant method for directed ESC differentiation.

### Conclusions

The present study describes the synthesis and characterization of electrospun fibers comprised of a subset of a library of polymers with different physicochemical properties. Among the polymers tested, the polymer composition of 4%PEG-86%PCL-10%CPCL optimally facilitated the differentiation of ESCs into functional cardiomyocytes. This effect correlated with the local density of deposited fibers, demonstrating a relationship between substrate mechanical properties and cell differentiation. The resulting cardiomyocytes were electrophysiologically functional, possessing desired Ca^2+^ depolarization and repolarization pathways. These results are promising for the treatment of cardiac ischemia, where the ensuing myocardial hypoxia, necrosis, and fibrosis may be treated through the delivery of a tissue-engineered myocardial patch. The methods described provide an efficient way to induce functional cardiomyocytes from pure ESCs, which is of great interest to the areas of regenerative medicine biomaterials, tissue engineering, cardiology, and stem cell biology.
