# Correction: Combinatorial Polymer Electrospun Matrices Promote Physiologically-Relevant Cardiomyogenic Stem Cell Differentiation

**DOI:** 10.1371/annotation/98390aa9-7a14-4b16-bbf9-da5322752b00

**Published:** 2012-09-14

**Authors:** Mukesh K. Gupta, Joel M. Walthall, Raghav Venkataraman, Spencer W. Crowder, Dae Kwang Jung, Shann S. Yu, Tromondae K. Feaster, Xintong Wang, Todd D. Giorgio, Charles C. Hong, Franz J. Baudenbacher, Antonis K. Hatzopoulos, Hak-Joon Sung

One of the affiliations of Charles C. Hong was erroneously excluded. Charles C. Hong is also affiliated with Research Medicine, Veterans Affairs TVHS, Nashville, Tennessee. 

